# Intracellular glycolysis in brown adipose tissue is essential for optogenetically induced nonshivering thermogenesis in mice

**DOI:** 10.1038/s41598-018-25265-3

**Published:** 2018-04-27

**Authors:** Jae Hoon Jeong, Ji Suk Chang, Young-Hwan Jo

**Affiliations:** 1 Division of Endocrinology, Department of Medicine, Albert Einstein College of Medicine, 1300 Morris Park Avenue, Bronx, NY 10461 USA; 2 Department of Molecular Pharmacology, Albert Einstein College of Medicine, 1300 Morris Park Avenue, Bronx, NY 10461 USA; 3 Laboratory of Gene Regulation and Metabolism, Pennington Biomedical Research Center, 6400 Perkins Road, Baton Rouge, LA 70808 USA

## Abstract

Release of fatty acids from lipid droplets upon activation of the sympathetic nervous system (SNS) is a key step in nonshivering thermogenesis in brown adipose tissue (BAT). However, intracellular lipolysis appears not to be critical for cold-induced thermogenesis. As activation of the SNS increases glucose uptake, we studied whether intracellular glycolysis plays a role in BAT thermogenesis. To stimulate BAT-innervating sympathetic nerves *in vivo*, we expressed channelrhodopsin-2 (ChR2) in catecholaminergic fibers by crossbreeding tyrosine hydroxylase-Cre mice with floxed-stop ChR2 mice. Acute optogenetic stimulation of sympathetic efferent fibers of BAT increased body temperature and lowered blood glucose levels that were completely abolished by the β-adrenergic receptor antagonist. Knockdown of the *Ucp1* gene in BAT blocked the effects of optogenetic stimulation on body temperature and glucose uptake. Inhibition of glucose uptake in BAT and glycolysis abolished optogenetically induced thermogenesis. Stimulation of sympathetic nerves upregulated expression of the lactate dehydrogenase-A and -B genes in BAT. Optogenetic stimulation failed to induce thermogenesis following treatment with the LDH inhibitor. Pharmacological blockade and genetic deletion of the monocarboxylate transporter 1 completely abolished the effects of sympathetic activation. Our results suggest that intracellular glycolysis and lactate shuttle play an important role in regulating acute thermogenesis in BAT.

## Introduction

Interscapular brown adipose tissue (BAT) is the main organ that generates heat through nonshivering thermogenesis. BAT contains a high density of mitochondria with uncoupling protein-1 (UCP-1) that uncouples mitochondrial oxidative respiration from ATP production to produce heat^1–4^. BAT also possesses a great capacity for glucose uptake and utilization^5^. Cold exposure increases glucose uptake by BAT in rodents as well as humans^6–9^. Although the function of the parasympathetic nervous system in BAT remains elusive, nonshivering thermogenesis and glucose uptake by BAT are regulated by the sympathetic nervous system (SNS)^5,10^. For instance, mice lacking the β-adrenergic receptors are cold intolerant^11^ and activation of the β3-adrenergic receptor (β3AR) in BAT stimulates glucose uptake^12–15^. The ability of BAT for glucose clearance makes this tissue a therapeutic potential target for reducing blood glucose levels in humans^10,16,17^.

Intracellular lipolysis and mitochondrial fatty acid β-oxidation appear to be essential for producing a protonmotive force that drives the proton leak through UCP1^3,5^. In other words, activation of the β3AR stimulates intracellular lipolysis and then free long chain (LC) fatty acids released from lipid droplets are transported to mitochondria via carnitine palmitoyltransferase 1 and 2 (CPT1 and 2)^2,3^. Mitochondrial fatty acid β-oxidation generates acetyl-CoA that feeds the tricarboxylic acid (TCA) cycle. Disruption of mitochondrial β-oxidation blocks nonshivering thermogenesis^18–20^. For instance, mice lacking the *Cpt2* gene in adipocytes are cold-intolerant^18^. BALB/cByJ mice having a mutated gene encoding the short chain acyl CoA dehydrogenase are sensitive to cold^20^ and mice carrying the targeted inactivation of the LC acyl CoA dehydrogenase gene (*Acadl*) are also sensitive to a cold-challenge^20^. In addition, fatty acids directly bind and activate UCP1 that is generally blocked by purine nucleotides^21^. Therefore, the prior findings suggest the importance of β3AR-induced intracellular lipolysis in the control of UCP1-mediated heat generation in BAT.

However, two recent studies describe that intracellular lipolysis in BAT appears not to be critical in cold-induced nonshivering thermogenesis^22,23^. In mice with impaired adipose triglyceride lipase (ATGL)- and comparative gene identification-58 (CGI-58)-mediated lipolysis, nonshivering thermogenesis appears to depend on nutrient supply such as circulating glucose and fatty acids possibly from white adipose tissue^22,23^. As BAT is able to uptake large amounts of circulating glucose upon activation of the β3AR, glucose taken up would contribute to nonshivering thermogenesis as well. Indeed, early studies show that glucose oxidation contributes to nonshivering thermogenesis in rodents, although only a small portion of glucose taken up is used for thermogenesis^24,25^. In addition, prevention of glucose uptake in mouse BAT severely impairs nonshivering thermogenesis^26^, suggesting the importance of glucose in BAT thermogenesis.

Rabinowitz and colleagues recently demonstrate that lactate is a primary TCA substrate in all tissues, including adipose tissue^27^. Interestingly, a study in the late 1960s and a recent study describes that activation of β-adrenergic receptors in BAT converts glucose to lactate in brown adipocytes^28,29^. Based on these prior findings, we hypothesized that lactate from intracellular glycolysis is an alternative fuel substrate for BAT thermogenesis. To examine the contribution of intracellular glycolysis to BAT thermogenesis, we expressed light-activated channelrhodopsin-2 (ChR2) in catecholaminergic fibers by crossbreeding tyrosine hydroxylase-Cre mice with floxed-stop ChR2 mice and optogenetically stimulated sympathetic efferent fibers innervating BAT. We found that 1 hr optogenetic stimulation of sympathetic nerves of BAT increased body and BAT temperature that required intracellular glycolysis in BAT.

## Results

### Optogenetic stimulation of sympathetic efferent fibers of BAT elevates BAT temperature and lowers blood glucose level

**Figure 1:**
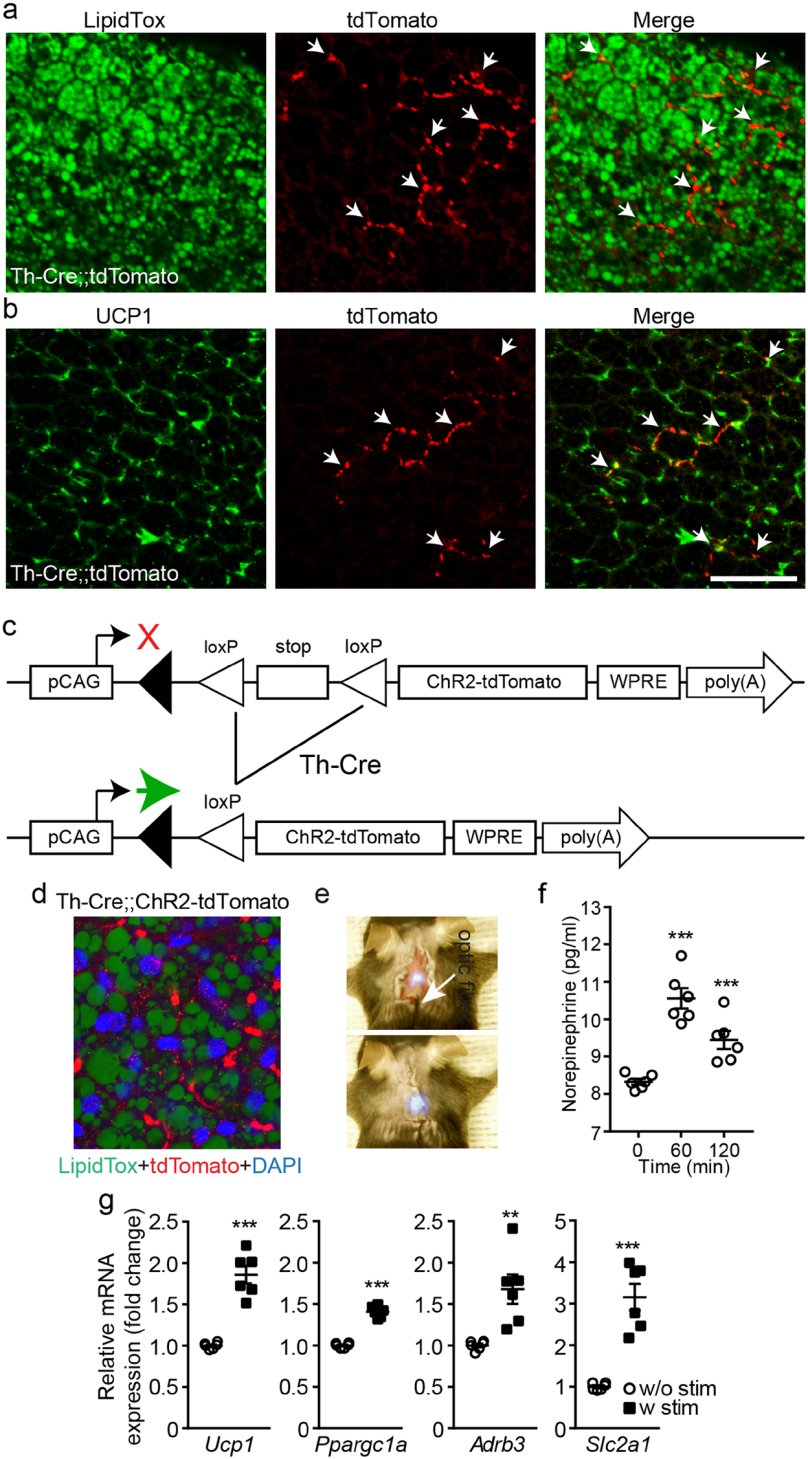
Optogenetic stimulation of sympathetic efferent fibers innervating BAT upregulates thermogenic gene expression. (**a** and **b**) Images of confocal fluorescence microscopy showing double immunostaining of BAT in Th-Cre;;tdTomato mice with LipidTox (green, A) anti-UCP1 (green, B) and anti-tdTomato (red) antibodies. Arrowheads represent TdTomato-positive efferent fibers in the BAT parenchyma. Scale bar: 50 μM. (**c**) Schematic diagram of our breeding strategy to generate Th-Cre;;ChR2-tdTomato mice. (**d**) Image of confocal fluorescence microscopy showing triple immunostaining of BAT in Th-Cre;;ChR2-tdTomato mice with LipidTox (green), anti-tdTomato (red), and DAPI (blue) antibodies. (**e**) Images showing our experimental configuration. An optic fiber was directly placed underneath the BAT pad and then the pad was again covered with the skin. (**f**) Pooled data showing NE release following optogenetic stimulation of sympathetic nerves of BAT (n = 6 mice). ***P < 0.001 (unpaired *t*-test). Data are shown as mean ± SEM. (**g**) Pooled data showing changes in *Ucp1*, *Ppargc1a* (*Pgc1a*), *Adrb3* (β*3AR*), *Slc2a1* (*Glut1*) mRNA expression levels with and without optogenetic stimulation of sympathetic nerves of BAT (n = 6 mice, respectively, unpaired *t*-test). w/o: without, w- with **p < 0.01, ***p < 0.001 (unpaired test). Data are shown as mean ± SEM.

To examine whether brown adipocytes receive sympathetic efferent innervation in our preparations, we generated tyrosine hydroxylase (Th)-Cre;;TdTomato mice, wherein the Th-Cre transgene causes cell-specific recombination to induce expression of tdTomato. We found that there were tdTomato-positive fibers in the BAT parenchyma and that lipid droplet-containing cells were innervated by tdTomato-positive fibers (Fig. 1a). Immunohistochemical analysis with an anti-UCP1 antibody further showed that UCP1-positive cells received sympathetic axonal projections (Fig. 1b). To selectively stimulate these sympathetic efferent fibers of BAT, we expressed ChR2 in catecholaminergic neurons by crossbreeding Th-Cre mice with floxed-stop ChR2-tdTomato mice (Th-Cre;;ChR2, Fig. 1c). Positive immunostaining for tdTomato confirmed the successful introduction of the ChR2 construct into sympathetic nerve endings innervating BAT (Fig. 1d).

An optic fiber was directly placed underneath the BAT pad of Th-Cre;;ChR2 mice (Fig. 1e). We first examined whether optogenetic stimulation of ChR2-expressing sympathetic nerve fibers of BAT elevated norepinephrine (NE) levels. Bursts of light pulses were applied for 1 s followed by a 1 s break that continuously repeated for 1 hr. The burst frequency was 20 Hz. There was a significant elevation in NE levels after optogenetic stimulation (Fig. 1f). We then investigated that optogenetic stimulation of sympathetic nerves regulates thermogenic gene expression in BAT. This optogenetic stimulation significantly increased expression levels of *Ucp1*, *Ppargc1a* (*Pgc1α*), and *Adrb3* (β3AR) mRNA (Fig. 1g). In particular, *Slc2a1* (*Glut1*) mRNA expression was strongly upregulated by this optogenetic stimulation (Fig. 1g). Our results indicate that short-term optogenetic stimulation of sympathetic efferent fibers of BAT was sufficient to increase thermogenic gene expression in BAT.

**Figure 2:**
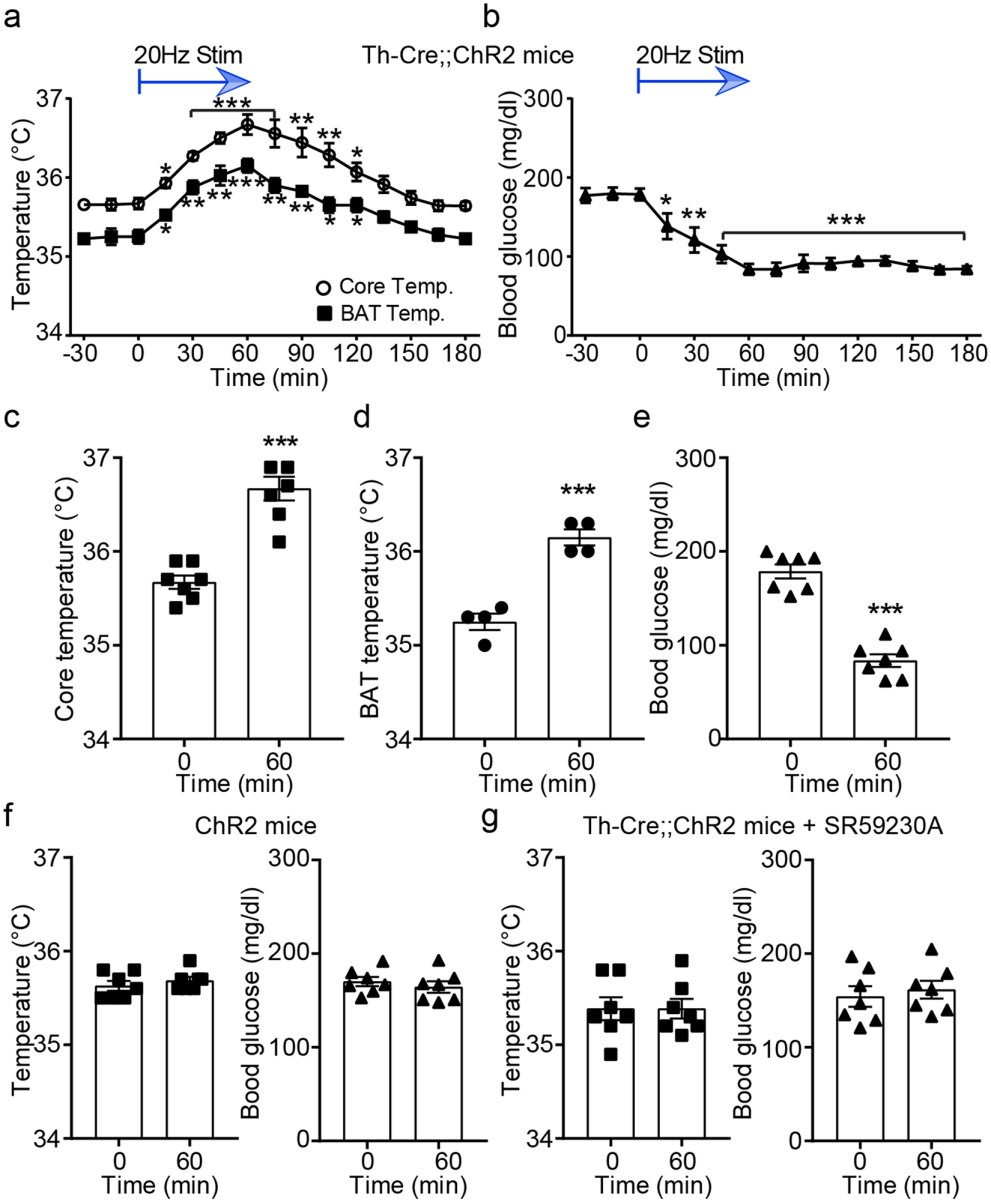
Optogenetic stimulation of sympathetic efferents of BAT increases body temperature but lowers blood glucose levels. (**a**) Pooled data showing that optogenetic stimulation of sympathetic efferent fibers of BAT increased body core (open circle; n = 7 mice) as well as BAT (filled square; n = 4 mice) temperature. The maximum increase was observed at the end of stimulation (at 60 min). *p < 0.05, **p < 0.01, ***p < 0.001 (ANOVA test). Data are shown as mean ± SEM. (**b**) Pooled data showing reduction in blood glucose levels during optogenetic stimulation of sympathetic innervation (n = 7 mice). *p < 0.05, **p < 0.01, ***p < 0.001 (ANOVA test). Data are shown as mean ± SEM. (**c** and **d**) Plots showing individual data as to body core (filled square) and BAT (filled circle) temperature at 0 and 60 min (***p < 0.001, unpaired *t*-test). Data are shown as mean ± SEM. (**e**) Plots showing individual data as to blood glucose levels (filled triangle) at 0 and 60 min (***p < 0.001, unpaired *t*-test). Data are shown as mean ± SEM. (**f**) Plots showing individual data as to body core temperature (left panel) and blood glucose levels (right panel) in control mice (ChR2 mice) at 0 and 60 min (n = 7 mice). Data are shown as mean ± SEM. (**g**) Pooled data showing the effects of the β3 receptor antagonist SR59230A (1 μM, n = 7 mice). SR59230A was directly injected into the BAT pad of Th-Cre::ChR2 mice. Under these experimental conditions, optogenetic stimulation did not increase body core temperature and glucose uptake by BAT. Data are shown as mean ± SEM.

We then investigated whether optogenetic stimulation of sympathetic efferent fibers is able to elevate BAT as well as body temperature. We found that the same optogenetic stimulation significantly increased both BAT and body temperature (Fig. 2a,c,d). Increased temperature returned to baseline levels within 2 hr (Fig. 2a). In contrast, control mice (i.e. ChR2 mice) did not respond to stimulation of sympathetic efferents (Fig. 2f). Of particular interest was that increased body temperature was always associated with a robust reduction in blood glucose levels (Fig. 2b,e). This effect lasted for more than 2 hr. In another set of experiments, we injected a retrograde viral vector carrying a Cre-dependent ChR2 expression cassette directly into BAT of Th-Cre mice (Supplementary Fig. 1). Optogenetically induced effects were similar to those observed in Th-Cre;;ChR2 mice under these experimental conditions, further supporting the interpretation that our approach stimulates sympathetic efferent fibers innervating BAT. Therefore, our results support the interpretation that, like cold-exposure and treatment with the β3AR agonists in rodents and humans^12–15,30–34^, 1 hr optogenetic stimulation of sympathetic efferent fibers of BAT increases body temperature and lowers blood glucose levels.

We next examined whether the effects of optogenetic stimulation on body temperature and blood glucose levels are due to activation of β-adrenergic receptors expressed in BAT. We directly injected a β3AR antagonist into the BAT pad 30 min prior to optogenetic stimulation. Treatment with the β3AR antagonist (SR59230A, 1μM) completely abolished the effects of optogenetic stimulation on body temperature and blood glucose levels (Fig. 2g), consistent with the prior studies describing the involvement of the β3AR^7,8,10–14^. As there was a strong positive correlation between BAT and body temperature as described in our prior study^35^, we analyzed body temperature and blood glucose levels as functional readouts of sympathetic activation of BAT.

As blue light can readily penetrate human and rodent skin, we took advantage of this property to stimulate ChR2-expressing sympathetic efferent fibers without any surgery (see Materials and Methods). We directly applied light through the skin of the mice with 2 mm × 2 mm SMD-LED modules (Supplementary Fig. 2a). ChR2-expressing sympathetic nerves of BAT were illuminated with bursts of light pulses for 1 hr. Likewise, this non-invasive optogenetic stimulation method was sufficient to raise body temperature and glucose uptake by BAT (Supplementary Fig. 2b,c). Therefore, our results suggest that both conventional and non-invasive optogenetic stimulation methods have the capability to stimulate sympathetic nerves of BAT, resulting in nonshivering thermogenic responses and glucose uptake by BAT.

### UCP1 plays a role in optogenetic stimulation-induced glucose uptake in BAT

**Figure 3:**
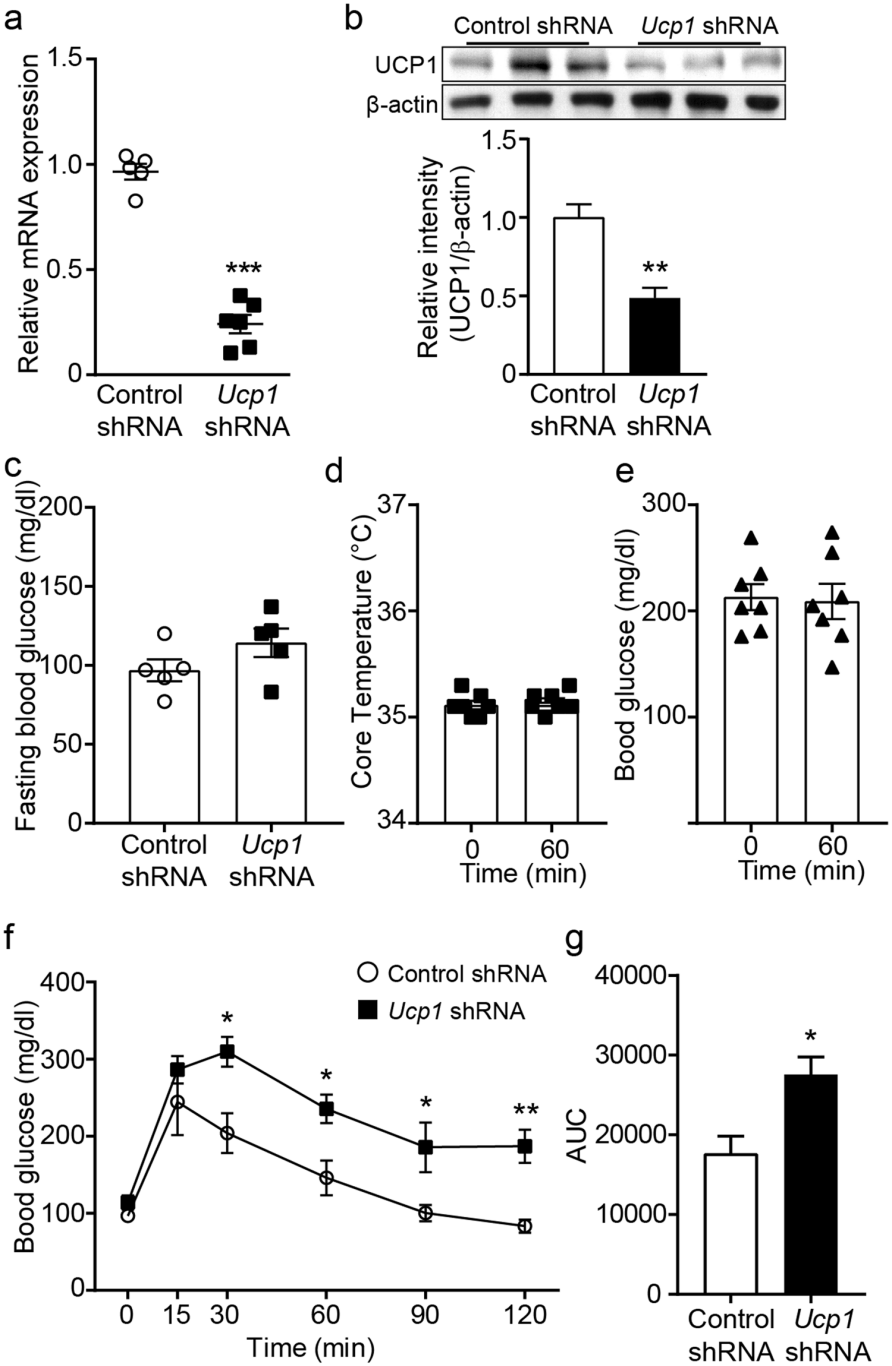
Glucose uptake requires UCP1 in BAT. (**a**) Plot showing reduced *Ucp1* mRNA expression in mice injected with *Ucp1* shRNA into the BAT pad compared with that in mice injected control shRNA (n = 5 and 6 mice, ***p < 0.001, unpaired *t*-test). Data are shown as mean ± SEM. (**b**) Western blotting to show knockdown of the *Ucp1* gene in BAT injected with *Ucp1* shRNA. Cropped images of western blotting showing knockdown of UCP1 protein (upper panel). Plot showing relative expression of UCP1 protein (bottom panel, **p < 0.01, unpaired *t*-test). Data are shown as mean ± SEM. (**c**) Plot showing baseline fasting blood glucose levels in mice injected with control or *Ucp1* shRNA into BAT (n = 5 mice, respectively). Data are shown as mean ± SEM. (**d** and **e)**. Pooled data from 7 mice showing that optogenetic stimulation failed to increase body temperature and lower blood glucose levels in mice injected with *Ucp1* shRNA into the BAT pad. Data are shown as mean ± SEM. (**f** and **g)**. Pooled data from 5 mice showing impaired OGTT in mice injected with *Ucp1* shRNA into BAT (f, *p < 0.05, **p < 0.01, unpaired *t*-test). Integrated glucose AUC over first 120 minutes of OGTT (g, *p < 0.05, unpaired *t*-test). Data are shown as mean ± SEM.

There has been controversy over whether β3AR-induced glucose uptake in BAT is UCP1-dependent. In fact, NE-induced glucose uptake appears to require UCP1 in BAT as UCP1 KOs show no stimulatory effect of NE on 2DG uptake^36^. In contrast to these findings, it has also been described that cultured mature brown adipocytes treated with *Ucp*1 siRNA and brown adipocytes from UCP1 KO mice retain glucose uptake in response to NE and β3AR agonists^29,31,37,38^. We thus examined whether optogenetically induced glucose uptake involves UCP1 uncoupling. We bilaterally injected *Ucp1* shRNA to the BAT pad of Th-Cre;;ChR2 mice. At two weeks post shRNA injections, mice injected with *Ucp1* shRNA showed a significant reduction in *Ucp1* mRNA and protein expression compared with that observed in mice injected with control shRNA (Fig. 3a,b, and Supplementary Fig. 3). Under these experimental conditions, there was no significant difference in fasting glucose levels between mice injected with control shRNA and those injected with *Ucp1* shRNA (Fig. 3c).

We found that optogenetic stimulation of sympathetic efferent fibers of BAT in *Ucp1* shRNA-injected mice did not lead to an elevation in body temperature (Fig. 3d). Moreover, blood glucose levels remained constant during stimulation (Fig. 3e). Oral glucose tolerance test (OGTT) further revealed that mice injected with *Ucp1* shRNA in BAT exhibited the significantly impaired ability to lower blood glucose levels in response to exogenous glucose challenge (Fig. 3f,g). Taken together, optogenetically induced glucose uptake into BAT appears to be associated with UCP1-mediated thermogenesis.

### Intracellular glycolysis is required for optogenetically induced thermogenesis

**Figure 4:**
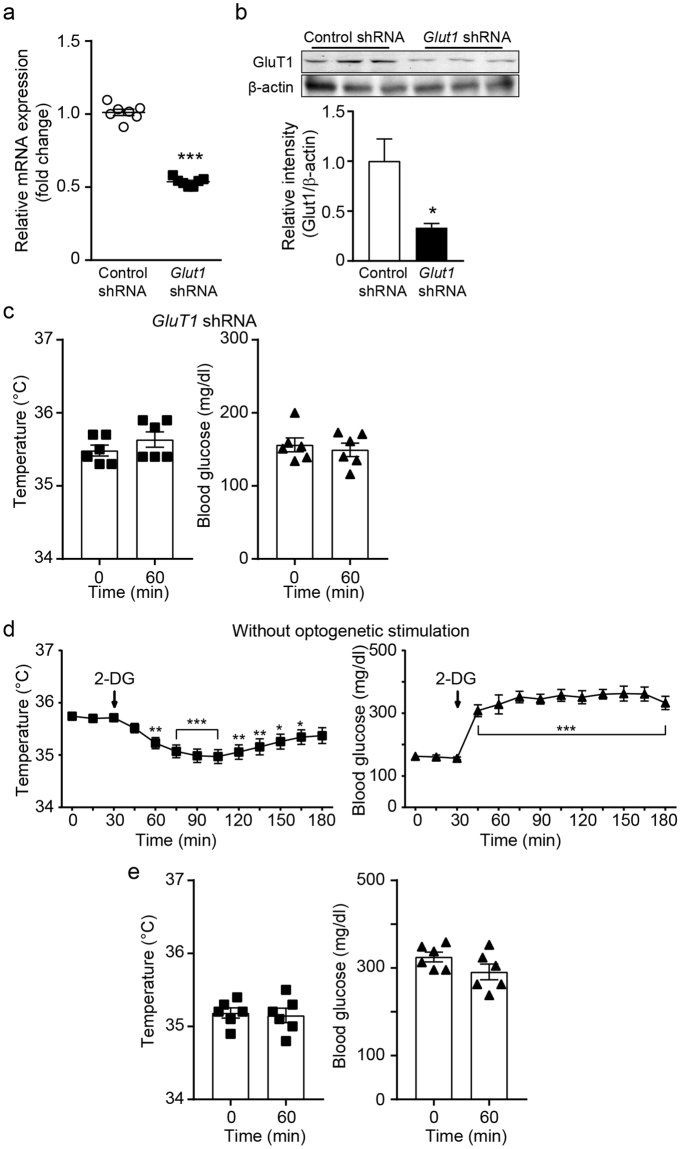
Glucose uptake and glycolysis are necessary for thermogenesis. (**a**) Plot showing relative *Slc2a1* mRNA expression in mice injected with control and *Slc2a1* shRNA into BAT (n = 7 mice, ***p < 0.001, unpaired *t*-test). Data are shown as mean ± SEM. (**b**) Western blotting to show knockdown of the *Glut1* gene in BAT injected with *Glut1* shRNA. Cropped images of western blotting showing knockdown of GLUT1 protein (upper panel). Plot showing relative expression of GLUT1 protein (bottom panel, *p < 0.05, unpaired *t*-test). Data are shown as mean ± SEM. (**c**) Pooled data from 6 mice lacking the *Slc2a1* (*Glut1)* gene in BAT. There was no change in body temperature and blood glucose levels during optogenetic stimulation of sympathetic nerves of BAT. Data are shown as mean ± SEM. (**d**) Pooled data showing effects of 2-DG on baseline body temperature and blood glucose levels. Subcutaneous injection of 2-DG reduced body core temperature, while increasing blood glucose levels (n = 7 mice). *p < 0.05, **p < 0.01, ***p < 0.001 (ANOVA test) Data are shown as mean ± SEM. (**e**) Pooled data showing effects of 2-DG. As baseline body temperature and blood glucose levels were stable 1 hr post 2-DG injection, sympathetic efferent fibers were stimulated 1 hr following 2-DG injection. Mice treated with 2-DG showed no response to optogenetic stimulation (n = 6 mice). Data are shown as mean ± SEM.

Circulating nutrients such as glucose and fatty acids appear to be sufficient to fuel BAT thermogenesis in mice lacking ATGL and CGI-58 in BAT^22,23^. To investigate whether circulating glucose uptake contributes to optogenetically induced thermogenesis, we silenced the *Slc2a1* (*Glut1)* gene in BAT as glucose transporter 1 (GLUT1) is a critical component in regulating NE-induced glucose uptake in BAT^12,13,39^. Mice injected with *Slc2a1* shRNA showed a significant reduction in *Slc2a1* mRNA and protein expression in BAT compared with that in mice injected with control shRNA (Fig. 4a,b, and Supplementary Fig. 3). Under these experimental conditions, optogenetic stimulation of sympathetic nerves failed to change body temperature and blood glucose levels in mice injected with *Slc2a1* shRNA (Fig. 4c), despite the fact that mitochondrial UCP1 can be directly activated by fatty acids^21,40–42^ and use fatty acids as a fuel source^3,5^.

We next investigated whether intracellular glycolysis of glucose taken up plays a role in optogenetically induced thermogenesis. We subcutaneously injected 2-deoxy-D-glucose (2-DG), a non-metabolizable glucose analogue (0.5 g/kg), over the neck of the mice. Administration of 2-DG alone significantly lowered body temperature, while elevating blood glucose levels (Fig. 4d) as described in the prior studies in rodents and humans^43,44^. Following stabilization of baseline body temperature and blood glucose levels (i.e. approximately 60 min post injections), optogenetic stimulation was performed. We found that inhibition of glycolysis with 2-DG completely abolished the effects of optogenetic stimulation on body temperature and blood glucose levels (Fig. 4e), suggesting that the importance of glycolysis in BAT thermogenesis.

**Figure 5:**
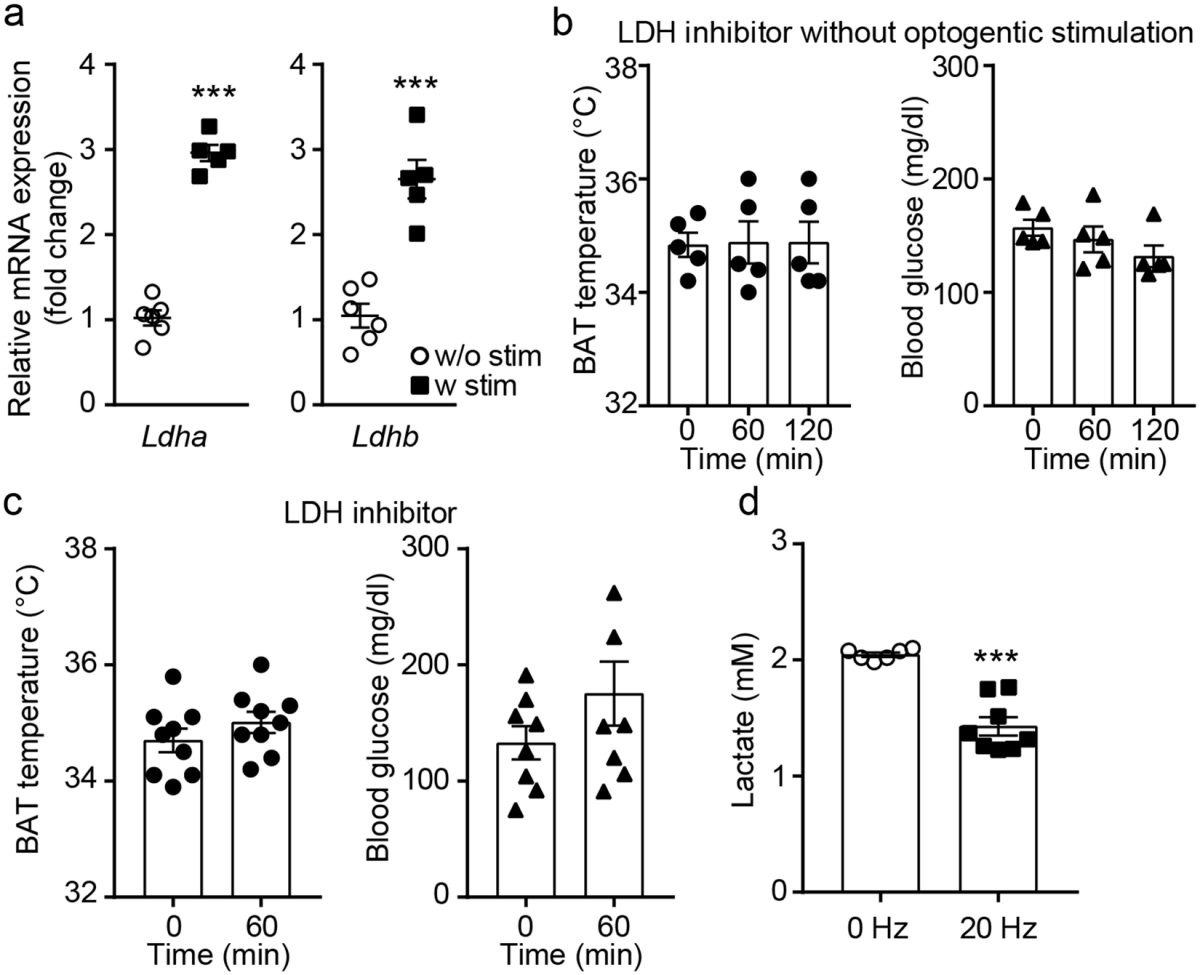
LDH activity is critical for BAT thermogenesis. (**a**) Pooled data showing increased *Ldha* and *Ldhb* genes upon optogenetic stimulation of sympathetic nerves of BAT (***p < 0.001, unpaired *t*-test). Data are shown as mean ± SEM. (**b**) Effects of the LDH inhibitor sodium oxamate on baseline body temperature and blood glucose levels. Data are shown as mean ± SEM. (**c**) Pooled data showing that mice treated with sodium oxamate did not respond to optogenetic stimulation of sympathetic efferents of BAT. Data are shown as mean ± SEM. (**d**) Pooled data showing lactate levels in BAT with or without optogenetic stimulation (***p < 0.001, unpaired *t*-test). Data are shown as mean ± SEM.

Pyruvate can be converted to lactate in the cytoplasm by lactate dehydrogenase (LDH). Thus, we examined whether expression of *Ldha* and *Ldhb* genes in BAT is regulated by 1 hr optogenetic stimulation. Optogenetic stimulation of sympathetic nerves significantly upregulated expression of *Ldha* and *Ldhb* mRNA in BAT (Fig. 5a), suggesting a potential role of LDH in the control of BAT thermogenesis. To investigate the importance of LDH activity in BAT thermogenesis, we directly injected the LDH inhibitor sodium oxamate^45^ into the BAT pad 1 hr prior to optogenetic stimulation. Without optogenetic stimulation, sodium oxamate (50 mM) did not alter baseline BAT temperature and blood glucose levels (Fig. 5b). In the presence of sodium oxamate, however, optogenetic stimulation of sympathetic nerves of BAT failed to increase BAT temperature and lower blood glucose levels (Fig. 5c), indicating that LDH activity is essential for acute activation of BAT thermogenesis.

We next examined whether there are changes in lactate levels in BAT during sympathetic activation. BAT was collected with or without 1 hr optogenetic stimulation. We found that lactate levels were significantly lower in BAT from mice with optogenetic stimulation than those observed in mice without optogenetic stimulation (Fig. 5d). Our findings may imply that lactate is transported to mitochondria and used as a fuel substrate.

### Intracellular lactate shuttle is important for BAT thermogenesis

**Figure 6:**
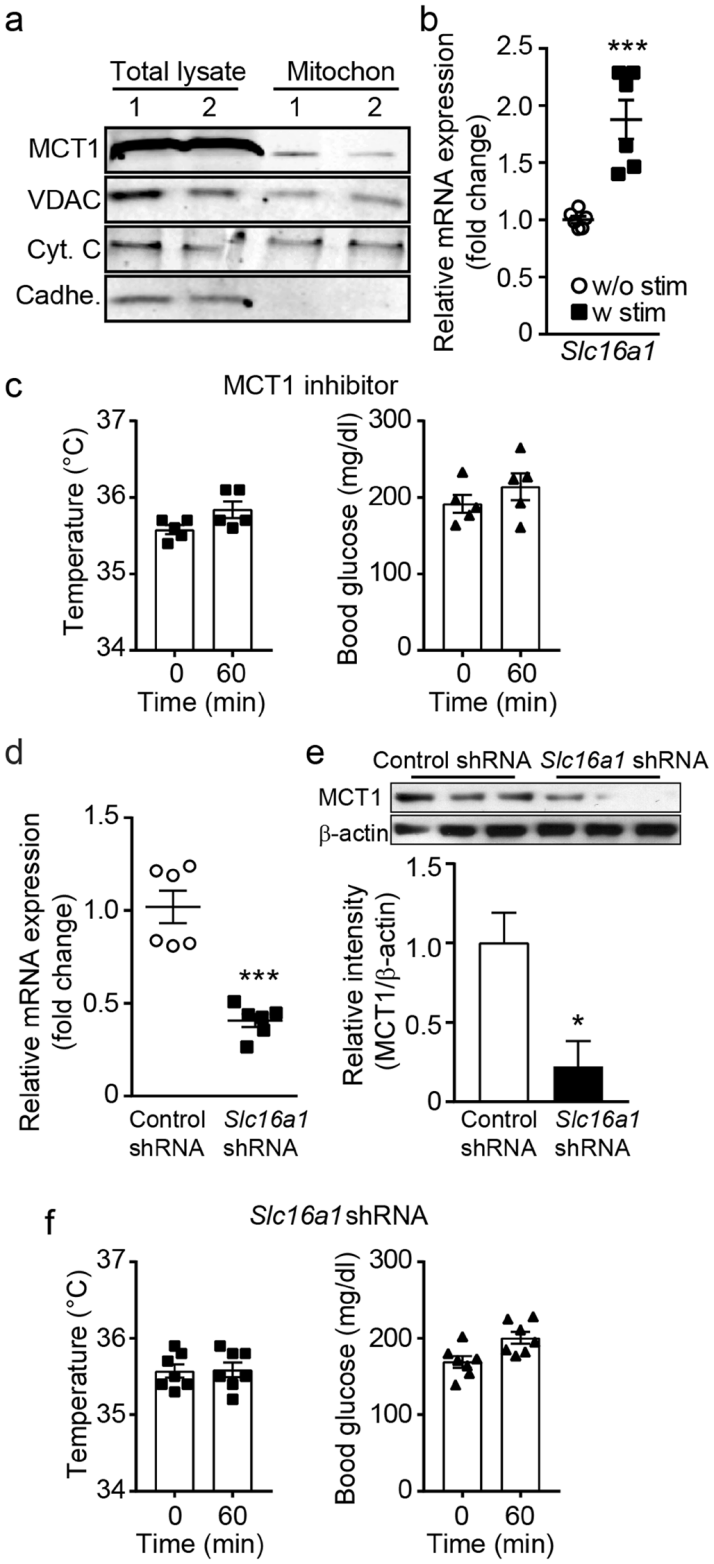
Blockade of MCT1 inhibits the effects of optogenetic stimulation of sympathetic efferent fibers of BAT. (**a**) Cropped images of western blotting showing expression of MCT1 in the mitochondrial fractions (n = 2 mice). Full-length blots are presented in Supplementary Figure 2. VDAC: voltage-dependent anion channel, Cyt. C: cytochrome C, Cadhe: Cadherin (**b**) Pooled data from 6 mice showing changes in *Slc16a1* (*Mct1*) mRNA expression with (filled square) and without (open circle) stimulation of sympathetic innervation of BAT (***p < 0.001, unpaired *t*-test). Data are shown as mean ± SEM. (**c**) Pooled data showing effects of blockade of the MCT1 on optogenetically induced increase in body temperature and glucose uptake (n = 5 mice). Data are shown as mean ± SEM. (**d**) Plot showing relative *Slc16a1 (Mct1)* mRNA expression in mice injected with control or *Slc16a1* shRNA into BAT (n = 6 mice, ***p < 0.001, unpaired *t*-test). Data are shown as mean ± SEM. (**e**) Western blotting to show knockdown of the *Mct1* gene in BAT injected with *Mct1* shRNA. Cropped images of western blotting showing knockdown of MCT1 protein (upper panel). Plot showing relative expression of MCT1 protein (bottom panel, *p < 0.05, unpaired *t*-test). Data are shown as mean ± SEM. (**f**) Pooled data showing that mice injected with *Slc16a1 (Mct1)* shRNA into the BAT pad showed no response to optogenetic stimulation. Data are shown as mean ± SEM.

As both pyruvate and lactate are transported to mitochondria via mitochondrial MCT1^45^, we examined whether BAT mitochondria express MCT1. Western blot analysis of total BAT lysates clearly revealed that MCT1 was abundantly expressed in BAT as described in the prior study^46^ (Fig. 6a and Supplementary Fig. 4). Importantly, MCT1 was also expressed in mitochondria (Fig. 6a and Supplementary Fig. 4). Purity of the isolated mitochondria was confirmed by the presence of mitochondrial membrane markers such as cytochrome C and voltage-dependent anion channel (VDAC), but no contamination with the transmembrane protein cadherin (Fig. 6a and Supplementary Fig. 4). In agreement with optogenetic induction of LDH expression in BAT, optogenetic stimulation of sympathetic innervation significantly upregulated expression levels of *Slc16a1* (*Mct1)* mRNA in Th-Cre::ChR2 mice (Fig. 6b).

We thus examined the effects of MCT1 activity on body temperature and blood glucose levels by direct injection of the MCT1 inhibitor (AR-C155858, 1 μM) into the BAT pad 30 min prior to optogenetic stimulation. Inhibition of MCT1 activity significantly blocked the effects of optogenetic stimulation (Fig. 6c). The importance of MCT1 activity in BAT thermogenesis and glucose uptake was further supported by experiments with knockdown of the *Slc16a1 (Mct1)* gene in BAT. At two weeks post *Slc16a1* shRNA injections to BAT, *Slc16a1* mRNA and protein levels were significantly downregulated in mice injected with *Slc16a1* shRNA compared with those in mice injected with control shRNA (Fig. 6d,e, and Supplementary Fig. 3). Under these experimental conditions, optogenetic stimulation of sympathetic efferent fibers failed to increase body temperature and lower blood glucose levels in mice injected with *Slc16a1* shRNA in BAT (Fig. 6f). Taken together, these findings suggest that lactate shuttle plays a critical role in regulating optogenetically induced BAT thermogenesis (Supplementary Fig. 5).

## Discussion

Our current study provides cellular evidence that intracellular glycolysis plays a role in the control of short-term nonshivering thermogenesis. 1 hr optogenetic stimulation of sympathetic efferent fibers innervating BAT was sufficient to generate nonshivering thermogenesis in Th-Cre;;ChR2 mice. Importantly, optogenetically induced nonshivering thermogenesis was always associated with a strong reduction in circulating glucose levels, consistent with the prior findings that stimulation of the β3AR in BAT increased glucose uptake and nonshivering thermogenesis (for review see^2,3,5,47^). Optogenetic stimulation-induced glucose uptake by BAT required UCP1 uncoupling as mice injected with *Ucp1* shRNA showed no change in blood glucose levels upon stimulation of sympathetic nerves. Importantly, inhibition of glucose uptake by knockdown of the *Glut1* gene in BAT and blockade of intracellular glycolysis with 2-DG completely abolished the effects of optogenetic stimulation. Interestingly, elevated sympathetic nerve activity upregulated LDH gene expression and blockade of LDH exclusively in BAT inhibited the effects of optogenetic stimulation. Furthermore, the MCT1 that transports both pyruvate and lactate was expressed in the mitochondria membrane and its blockade completely abolished the effects of sympathetic activation of BAT. Therefore, our results support the interpretation that intracellular glycolysis and lactate shuttle play a role in optogenetically induced nonshivering thermogenesis in BAT.

Although cold-exposure and/or β3AR agonists have been largely used to induce nonshivering thermogenesis, these methods could activate the SNS of not only BAT but also other peripheral organs due to hormonal changes. To circumvent those experimental limitations, we used optogenetics to stimulate ChR2-expressing sympathetic efferent fibers innervating BAT in Th-Cre;;ChR2 mice. A similar optogenetic approach was used to stimulate sympathetic inputs to white adipose tissues^48^. The major advantage of our optogenetic stimulation method was that we could manipulate BAT-innervating sympathetic nerves *in vivo* with high temporal resolution helping us examine the relationship between nonshivering thermogenesis and BAT-mediated glucose uptake. As selective stimulation of sympathetic innervation of BAT elevated body temperature and glucose uptake, the results obtained with our new approach were consistent with the prior findings describing the important role of the SNS in the control of nonshivering thermogenesis and glucose uptake in rodents and humans^12–15,30–34^. Our findings also raised a question as to the relative contribution of circulating glucose, lipids, and/or intracellular triglycerides as fuel substrates in particular to short-term nonshivering thermogenesis.

It has been thought that intracellular triglycerides were the main source of energy for nonshivering thermogenesis in rodents and humans^6,49^. In other words, NE-induced lipolysis and fatty acid β-oxidation appear to be critical for the production of a protonmotive force that catalyzes UCP1 uncoupling^3,5^. In fact, CPT2 KO mice were cold-sensitive and were not able to upregulate thermogenic genes in response to β3AR agonists^18^. Mice carrying the targeted inactivation of the LC acyl CoA dehydrogenase gene were also cold-intolerant^20^. In addition, recent positron emission tomography (PET) imaging studies with ^11^C-acetate to assess oxidative metabolism demonstrated that intracellular lipolysis significantly contributed to BAT thermogenesis in rats as well as humans^50,51^, further supporting the key role of intracellular lipid in BAT thermogenesis.

In contrast, recent studies with mice lacking ATGL or CGI-58 in BAT demonstrated that ATGL-/CGI-58-mediated intracellular lipolysis in BAT played a minor role in nonshivering thermogenesis^22,23^. In fact, mice defective in ATGL-/CGI-58-mediated lipolysis in BAT were cold-tolerant when food was present^22,23^. Thus, it appears that circulating nutrients are sufficient to generate nonshivering thermogenesis^22,23^. Interestingly, extensive proteomic studies of BAT demonstrated that levels of enzymes of the mitochondrial β-oxidation pathway were not changed during nonshivering thermogenesis^52^. Additionally, short-term cold-exposure did not increase the expression of the *Cpt1b* gene in BAT^51^. Therefore, it is plausible that BAT can use alternative thermogenic pathways in certain conditions such acute cold-exposure.

As the mitochondrial tricarboxylic acid (TCA) cycle is shared by both glucose and fatty acid metabolism, intracellular glycolysis may also contribute to nonshivering thermogenesis as described in the early study^53^. In fact, mice lacking mTORC2 in adipose tissues showed cold sensitivity that was due to impaired cold-induced glucose uptake in BAT and restoration of glucose uptake in BAT improved cold tolerance^26^. In line with this prior study, blockade of glucose uptake by knockdown of the *Glut1* gene in BAT completely abolished the effects of 1 hr optogenetic stimulation on body temperature and glucose uptake in our preparations, suggesting that nonshivering thermogenesis appears to require glucose uptake. However, it remains unclear whether NE-induced glucose uptake in BAT requires increased UCP-1 activity. For instance, NE increased glucose transport via mitochondrial thermogenesis in isolated brown adipocytes^30^ and UCP1 KO mice were no longer able to take up glucose in response to the β3AR activation^36^. Importantly, it has also been reported that brown adipocytes from UCP1 KO mice had the ability to take up glucose following activation of β3ARs^31,37,38^. Although there is no clear explanation for this discrepancy, it appears to be a consequence of the different systems used (e.g. glucose uptake measurements *in vivo*, *ex vivo* or *in vitro*, sex, and fasting). In other words, our indirect measurement of glucose uptake in BAT may explain the controversy about the importance of UCP1 for glucose uptake between previous studies and our present one. Hence, further studies are needed to confirm our findings.

As relatively large amounts of glucose were taken up following activation of β3ARs, increased glucose metabolism and oxidation would be expected in BAT, resulting in generation of the reduced form of nicotinamide adenine dinucleotide (NADH). NADH played a key role in anaerobic glycolysis in the cytoplasm^45^. The relative availability or ratio of NAD and NADH appears to be a critical determinant in the production of lactate from pyruvate^54^. As both *Ldha* and *Ldhb* genes were upregulated by optogenetic stimulation in our preparations, it was expected conversion of glucose to lactate upon activation of β3AR in BAT as described in the early study^28^. Interestingly, inhibition of LDH activity in BAT did not change baseline temperature and blood glucose levels. Thus, it seems likely that lactate production was minimal in a thermogenically quiescent state. However, optogenetic stimulation failed to elevate BAT temperature and lower blood glucose levels in mice treated with the LDH inhibitor, suggesting the importance of LDH activity in NE-induced thermogenesis.

Interestingly, lactate levels in BAT were significantly reduced by sympathetic stimulation, suggesting that lactate would be used during BAT thermogenesis. In fact, BAT mitochondria membrane expressed the MCT1 and sympathetic stimulation upregulated its mRNA expression in our preparations. These findings suggest that lactate can be transported to mitochondria in BAT. Interestingly, a recent study described that lactate was a major substrate for the TCA cycle in all the tissues, including adipocytes^27^. Thus, it seems likely that lactate transported to mitochondria also fed the TCA cycle in our preparations. In this case, lactate shuttle would produce the reduced hydrogen carriers that donate electrons to the electron transporter chain (ETC), which results in generation of a protonmotive force. It is plausible that BAT may use two independent thermogenic pathways (lipolytic versus glycolytic) depending on the degree of sympathetic activity of BAT. In fact, short-term cold-exposure did not change the expression of the *Cpt1b* gene in BAT^51^. It is possible that glycolysis and intracellular lactate shuttle would be sufficient for acute thermogenesis, whereas mitochondrial fatty acid β-oxidation would be absolutely required for thermogenesis in long-term cold-exposed animals. In other words, the use of lipids by the brown adipocyte would become the preferential way to fuel thermogenesis and glucose would in large part serve for *de novo* lipogenesis in cold-adapted animals.

In summary, the fate and role of glucose in the control of nonshivering thermogenesis have not drawn too much attention, although BAT has a great capacity for glucose uptake. Our present results support the interpretation that intracellular glycolysis and lactate shuttle are important for optogenetically induced nonshivering thermogenesis. Thus, it is necessary to examine the importance of BAT glycolysis and lactate metabolism during cold-induced thermogenesis in the future study. It will be also important to know whether BAT-derived endocrine hormones such as fibroblast growth factor 21 (FGF21)^55,56^ would play a role in lowering blood glucose levels in our preparations. Our findings provide possible cellular mechanisms underlying hyperglycemia in people with type 2 diabetes as glucose but not fatty acid oxidative metabolism in BAT is impaired in type 2 diabetes^57^. In addition, our findings that optogenetics can be employed noninvasively would have profound therapeutic implications as optogenetic stimulation of sympathetic fibers in BAT can in principle be used to stimulate blood glucose clearance by BAT as described in the literature^2^.

## Materials and Methods

### Animals

All mouse care and experimental procedures were approved by the institutional animal care research advisory committee of the Albert Einstein College of Medicine. All experiments were performed in accordance with relevant guidelines and regulations. Mice used in experiments included Th-Cre (Stock# 008601), floxed tdTomato (Stock# 007909), and floxed-stop ChR2-tdTomato (Stock# 012567) transgenic mice (The Jackson Laboratory). Both male and female mice on a mixed C57BL6/129SVJ background were used for all experiments. Animals were housed in groups in cages under conditions of controlled temperature (22 °C) with a 12:12 hr light-dark cycle and fed a standard chow diet with *ad libitum* access to water.

### Optogenetic stimulation of postganglionic sympathetic efferent fibers of BAT

To optogenetically stimulate sympathetic efferent fibers of BAT, optic fibers (Doric Lenses, Inc.) were directly placed underneath the BAT pad. Optic fibers were coupled to a 473 nm DPSS laser (Lasergrow technologies). We also used a novel non-invasive optogenetic stimulation method to stimulate channelrhodopsin-expressing sympathetic efferents using surface-mounted-device light-emitting device modules (SMD-LEDs) as a light source. In this case, we directly placed SMD-LED modules on the skin of the interscapular region. Each SMD-LED module was connected to a TTL pulse generator (Doric lenses) through a BNC cable. The rationale of this method is that light can easily penetrate through the skin of the animals. Optogenetic experiments were performed under isoflurane anesthesia at room temperature (24 °C). A warm pad was used to maintain the animal’s body temperature during anesthesia. In some experiments as noted, we injected hEF1α-LS1L-hChR2(H134R)-mCherry viruses (MIT vector core) into BAT of Th-Cre mice to express ChR2 in catecholaminergic fibers innervating BAT.

### Immunohistochemistry

Mice were transcardially perfused with PBS. BAT was fixed overnight at 4 °C with 4% paraformaldehyde and was cut 50 μM sections with a vibratome. The sections were incubated with 5% bovine serum albumin (BSA) at room temperature and then with anti-UCP1 (1:1000, Santa Cruz biotechnology, Inc., sc-6529) and anti-DsRed (1:1000, Clontech laboratories, Inc., 632496) antibodies in 0.2% Triton X-100 overnight at 4 °C. Following incubation with primary antibodies, the sections were washed 3 times in PBS and incubated with Alexa 488 anti-goat IgG (1:500, A11055) and Alexa 568 anti-rabbit IgG (1:500, Thermo Fisher Scientific, Inc., A10042) for 2 hr at room temperature. For lipid staining, tissues were incubated with LipidTox Green neutral lipid stain (1:200, Thermo Fisher Scientific, Inc., H34475) for 30 min at room temperature. Tissues were then washed, dried, and mounted with VECTASHIELD mounting media. Images were acquired using a scanning confocal microscope.

### Bilateral injections of drugs and shRNAs into BAT

Replication-incompetent lentiviral particles (Millipore Sigma, Inc.) encoding *Ucp1* shRNA (1.8 × 10^7^ TU/ml, clone # TRCN0000437754), *Slc2a1* (*GluT1)* shRNA (2.1 × 10^7^ TU/ml, clone # TRCN0000305719), and *Slc16a1* (*Mct1)* shRNA (2 × 10^6^ TU/ml, clone# TRCN0000079545) were bilaterally injected into the BAT pad of Th-Cre::ChR2 mice using a Hamilton syringe (1 μl per site and 5 sites per pad). For pharmacological experiments, drugs (AR-C155858, 1 μM, Tocris bioscience; SR59230A, 1 μM, Millipore Sigma Inc; Sodium Oxamate, 50 mM, Chem Cruz, sc-215880) were bilaterally injected to the BAT pad at five loci (1 μl per locus).

### Quantitative Real-Time PCR analysis

For qRT-PCR analysis of *Ucp1*, *Ppargc1a* (*Pgc1α*), *Adrb3* (β*3AR*), *Slc2a1* (*Glut1*), and *Slc16a1* (*Mct1*), *Ldha*, and *Ldhb* genes, total RNA was isolated using the Trizol reagent (Thermo Fisher Scientific, Inc., 15596026) from BAT and then first-strand cDNA was synthesized using SuperScript III First-Strand synthesis kit (Thermo Fisher Scientific, Inc., 18080-051). Real-time qPCR was performed in sealed 96-well plates with SYBR Green I master Mix using a Light Cycler 480 instrument (Roche Molecular Systems, Inc.). qPCR reactions were prepared in a final volume of 20 μl containing 2 μl cDNA and 10 μl of SYBR Green master mix in the presence of primers at 0.5 μM. β-2 microglobulin (B2M) was used as an internal control for quantification of each sample. A list of primer sets included: F5′-cgttccaggacccgagtcgcaga-3′ and R5′-tcagctcttgttgccgggttttg-3′ for *Ucp1*, F5′-gacagctttctgggtggatt-3′ and R5′-cgcaggctcattgttgtact-3′ for *Ppargc1a*, F5′-ggctctgtgtctctggttagt-3′ and R5′- gaggagacagggatgaaacct-3′ for *Adrb3*, F5′-ccatgtatgtgggagaggtg-3′ and R5′-ttgcccatgatggagtctaa-3′ for *Slc2a1*, F5′-aatgctgccctgtcctccta-3′ and R5′-cccagtacgtgtatttgtag-3′ for *Slc16a1*, F5′-tcgtgcactagcggtctcaa-3′ and R5′-aacagcaccaaccccaaca-3′ for *Ldha*, F5′-ggattcaccccgtgtctacc-3′ and R5′-gagcgacctcatcgtccttc-3′ for *Ldhb*, and F5′-ttcagtcgcggtcgcttc-3′ and R5′-aggccggtcagtgagacaag-3′ for *B2M*. Relative gene expression was determined using the ^ΔΔ^*Ct* method. Relative mRNA expression levels were presented as a fold change compared with those of the control group.

### Mitochondria Isolation and Western blotting

Whole cell lysates were prepared from BAT of Th-Cre::ChR2 mice using lysis buffer (25 mM Tris, 95 mM NaCl, 1% SDS, 1 mM EDTA, 1% protease inhibitor, and 1% phosphatase inhibitor). To detect knockdown efficiency by shRNA, the total lysates from BAT injected with control shRNA, *Mct1* shRNA, *GluT1*, and *Ucp1* shRNA were extracted 2 weeks post viral injections. Mitochondrial fractions were isolated with a mitochondria isolation kit (Thermo Fisher Scientific, Inc., 89801). Total lysates and mitochondrial fractions (25 μg each) were loaded in 10% SDS gels and transferred to the PVDF membrane (Thermo Fisher Scientific, Inc., IB24002). The membrane was incubated with blocking buffer (LI-COR, 927-40000) for 2 hr at room temperature and then with anti-MCT1 (1:1000, Novus Biologicals, NBP1–59656), anti-voltage-dependent anion channel (VDAC, 1:1000, Thermo Fisher Scientific, Inc., PA1-954A), anti-cytochrome C (1:1000, Thermo Fisher Scientific, Inc, 338200) and anti-pan Cadherin (1 μg/ml, Abcam, Inc., ab16505), anti-GluT1 (1:1000, Cell signaling, 12939), anti-UCP1 (1:500, Santa-Cruz, sc-6529), anti-β-actin (1:5000, Sigma-Aldrich, A5316) antibodies in TBST buffer containing 5% BSA for 24 hr at 4 °C. Following incubation with primary antibodies, the membrane was washed in TBST buffer, probed with IRDye 680LT Donkey anti-Mouse IgG (1:20000, LI-COR, 926-68022) and IRDye 800CW Donkey anti-Rabbit IgG (1:20000, LI-COR, 926-32213), and IRDye 680RD Donkey anti-Goat IgG (1:10000, Li-COR, 925-68074), and then visualized with the Odyssey CLx imaging system (LI-COR). The relative band intensity was measured using ImageJ software. β-actin was used to normalize changes in gene expressions.

### Measurement of body core/BAT temperature and blood glucose levels

Under anesthesia with isoflurane, we measured body core and BAT temperature at room temperature (24 °C). We inserted a wire thermoprobe (0.81 mm in diameter) into the rectum and a wire thermoprobe (0.23 mm in diameter, Physitemp Instruments, Inc.) underneath the BAT pad of Th-Cre::ChR2 mice as described in our prior study^35^. A warm pad was used to maintain the animal’s body temperature during anesthesia. We also collected blood from mouse tail every 15 min to measure blood glucose levels using a Accu-Chek Aviva Plus.

### Measurement of plasma NE levels

Under anesthesia with isoflurane, blood samples were collected into tubes with lithium heparin (VWR international, LLC) and centrifuged at 3000 rpm for 15 min to collect plasma. Plasma NE levels were quantified using a ELISA kit (MyBioSource, MBS701514).

### Oral glucose tolerance test and injection of 2-DG

For oral glucose tolerance test, 16 hr fasted mice received an oral glucose injection (2 g/kg) at time zero and blood glucose levels were monitored at 0, 15, 30, 60, 90, and 120 min. 2-deoxy-D-glucose (2-DG, 0.5 g/kg) was subcutaneously injected into the interscapular area 45 min prior to the optogenetic stimulation.

### Measurement of lactate from BAT lysate

Lactate levels from BAT lysate were measured with colorimetric L-Lactate Assay kit (Abcam, Inc., ab65331) according to the manufacturer’s instruction. BAT was collected with and without optogenetic-stimulation for 1 hr and then homogenized on ice. The assay was carried out with 5 ul of BAT lysates in a reaction mixture. Optical density of the samples was measured at a wavelength of 450 nm with SpectraMax Plus 384 microplate reader (Molecular Devices).

### Statistics

All statistics were performed with GraphPad Prism software. Data were expressed as mean ± SEM. Statistical significance was assessed using an unpaired *t*-test and multiple comparisons were tested with ANOVA followed by Bonferroni post-hoc analysis. The area under curve (AUC) for OGTT was calculated with GraphPad Prism software. Results with *p* < 0.05 were considered significant.

## Electronic supplementary material

Supplementary Figures
