# Supplementary material for: Intracellular glycolysis in brown adipose tissue is essential for optogenetically induced nonshivering thermogenesis in mice
Source: Sci Rep. 2018 Apr 27;8:6672. doi: 10.1038/s41598-018-25265-3 (PMC5923201; doi:10.1038/s41598-018-25265-3)
Supplement: Supplementary file 1 — Supplementary Figures [file 41598_2018_25265_MOESM1_ESM.pdf]

Supplementary Materials for

Intracellular glycolysis in brown adipose tissue is essential for optogenetically induced nonshivering thermogenesis in mice.

Jae Hoon Jeong, Ji Suk Chang, and Young-Hwan Jo

correspondence to: young-hwan.jo@einstein.yu.edu

This PDF file includes:

Supplementary Figures 1-5.

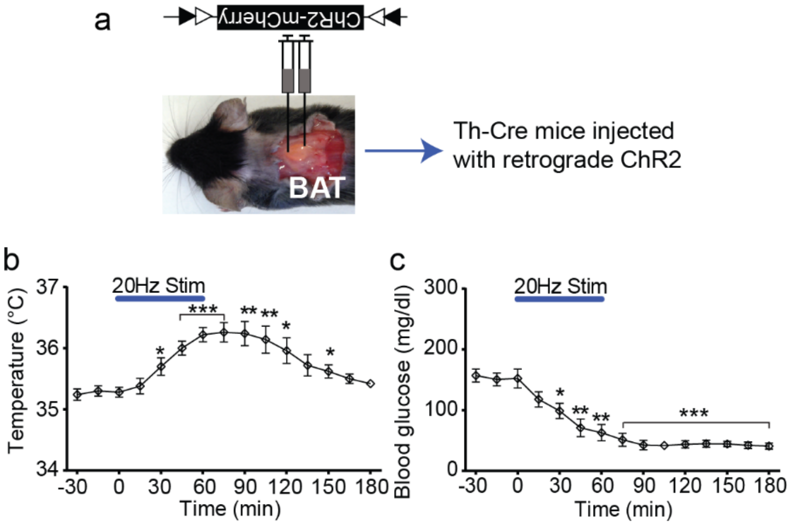

Supplementary Figure 1. Expression of ChR2 in Th-positive fibers innervating BAT.

a. Image showing our experimental configuration. Cre-dependent retrograde ChR2 viral vectors were injected into the BAT pad of Th-Cre mice.

b and c. Pooled data from 5 mice showing changes in body temperature and blood glucose levels in mice injected with the retrograde Cre-dependent ChR2 virus into BAT. Activation of sympathetic efferent fibers that exclusively innervate BAT not only elevated body temperature but also reduced blood glucose levels. Replication-incompetent herpes simplex viruses (hEF1α-LS1L-hChR2(H134R)-mCherry) were purchased from the MIT viral core.

All data are shown as mean ± SEM. *$p<0.05$; **$p<0.01$; ***$p<0.001$ (ANOVA test).

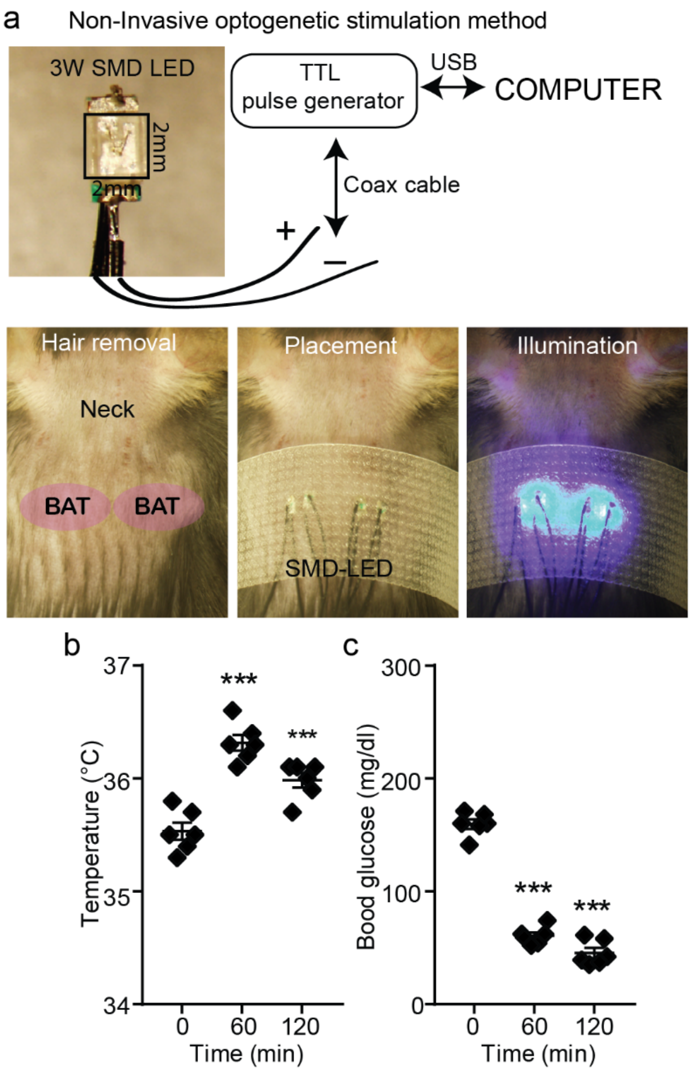

Supplementary Figure 2. Non-invasive optogenetic stimulation of sympathetic innervation of BAT.

a. Images showing our experimental configuration. 3W SMD-LED modules were controlled with a TTL pulse generator connected to a computer via USB. Each module was directly placed on the skin over the neck.

b and **c**. Pooled data showing changes in body temperature and blood glucose levels before, during, and after stimulation of sympathetic efferent fibers (n = 6 mice). ***p<0.001 (unpaired *t*-test)

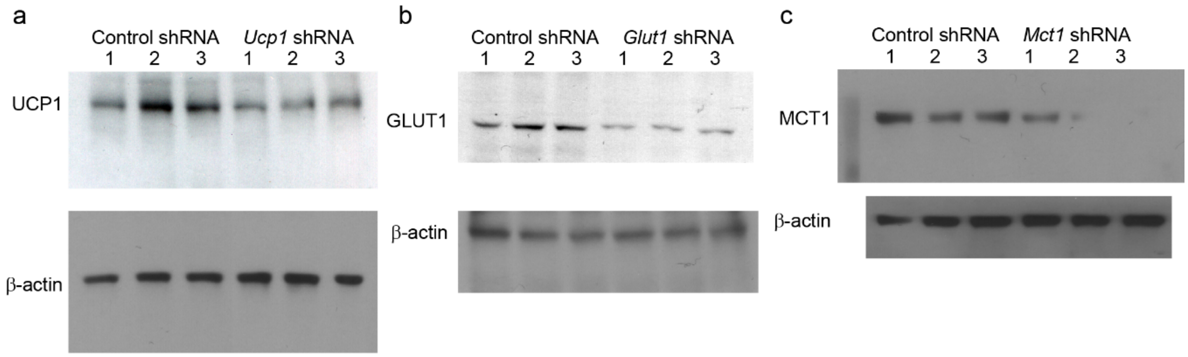

Supplementary Figure 3. Images of western blotting showing knockdown of *Ucp1 (a)*, *Glut1 (b)*, and *Mct1 (c)* genes in BAT.

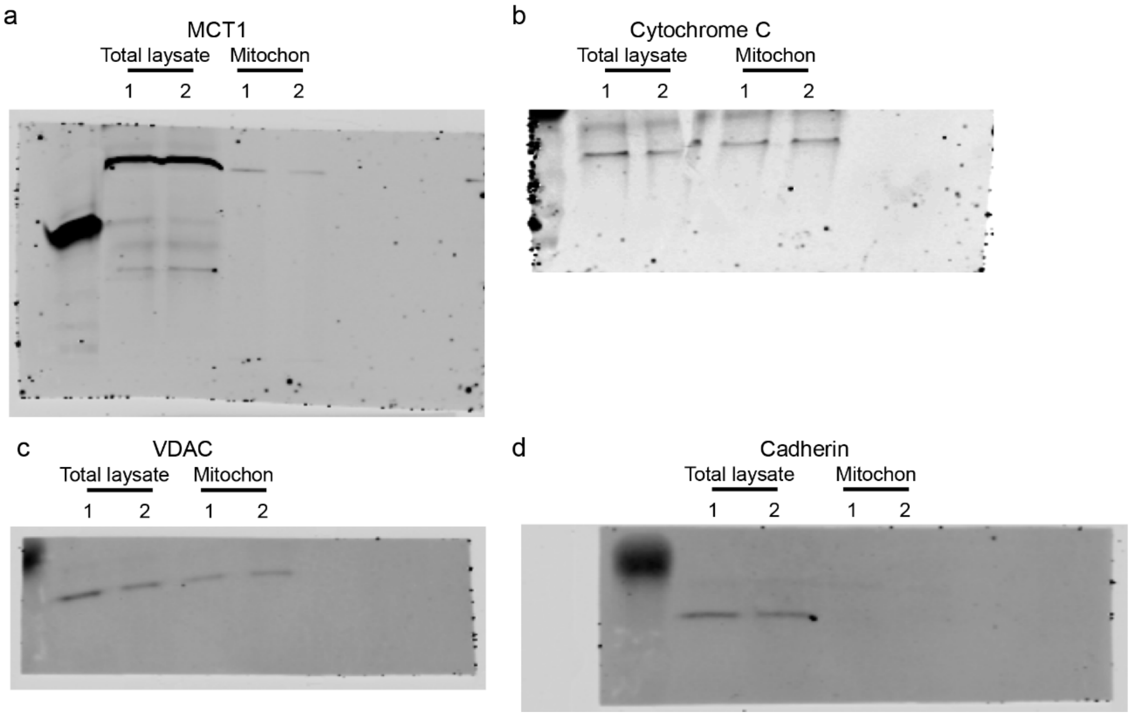

Supplementary Figure 4. Images of western blotting showing expression of MCT1 in the mitochondrial fractions. VDAC: voltage-dependent anion channel, Cyt. C: cytochrome C, Cadhe: Cadherin

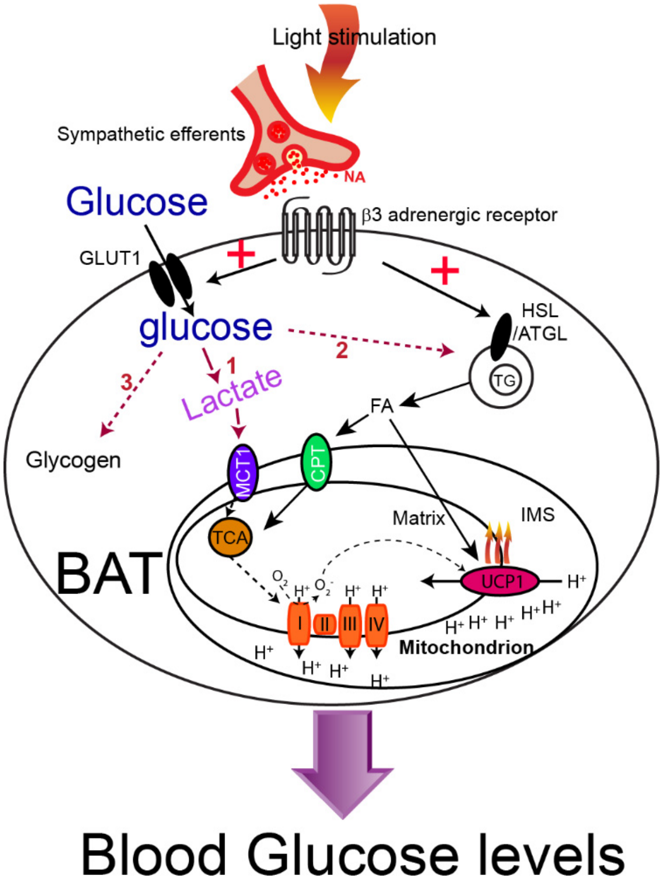

Supplementary Figure 5. Schematic illustration of the proposed model.

Activation of β3AR induces glucose uptake through GLUT1. As the mitochondrial TCA cycle is shared by both glucose and fatty acid metabolism, BAT can use both lipid and glucose to fuel nonshivering thermogenesis. As both *Ldha* and *Ldhb* genes are upregulated by optogenetic stimulation in our preparations, it is expected conversion of glucose to lactate upon activation of β3AR in BAT (1). Lactate is transported to mitochondria and feed the TCA cycle in BAT. Hence, it is possible that glycolysis and intracellular lactate shuttle would be sufficient for acute thermogenesis, whereas mitochondrial fatty acid β-oxidation would be absolutely required for thermogenesis in long-term cold-exposed and cold-adapted animals. In this case, glucose could in large part serve for *de novo* lipogenesis (2). Glucose can be used to produce glycogen (3).
